# Massachusetts

**Published:** 1896-08

**Authors:** 


*Massachusetts.* At Webster the herd of eleven kept at the poor-farms were tested in July, and nine were found diseased and destroyed. No other animals will be placed on the farm until after they are tested.

Sudbury farmers can hardly realize that their animals, so well apparently, could be the victims of tuberculosis, though the tuberculin-test revealed it and post-mortem confirmed the accuracy of the test.

